# Caudal regression syndrome type 1 with minimally invasive computed tomography and magnetic resonance imaging autopsy: a case report

**DOI:** 10.1186/s13256-023-04220-5

**Published:** 2023-11-25

**Authors:** Mira Ayoub, Chanae Dixon, Sharon E. Byrd, Rakhee M. Bowker

**Affiliations:** 1 Department of Diagnostic Radiology, Nuclear Medicine, Rush University Medical Center, Chicago, IL 60612 USA; 2 Division of Neonatology, Department of Pediatrics, Rush University Medical Center, 1653 West Congress Parkway, Suite 358 Pavilion, Chicago, IL 60612 USA

**Keywords:** Case report, Caudal regression syndrome, Minimally invasive postmortem, Fetal imaging, Computed tomography autopsy, Magnetic resonance imaging autopsy

## Abstract

**Background:**

Caudal regression syndrome is a rare complex congenital anomaly with reduced penetrance and phenotypic variability characterized by osseous defects of the caudal spine, lower limb anomalies, and accompanying genitourinary, gastrointestinal/anorectal, and cardiac system soft tissue defects. We report a rare presentation of type 1 caudal regression syndrome in a pregnant woman with preexisting diabetes, in which early recognition of severe fetal anomalies on routine antenatal ultrasound facilitated confirmation with fetal magnetic resonance imaging to characterize extent of disease and prognosticate fetal outcome.

**Case presentation:**

This case of type 1 caudal regression syndrome in the setting of maternal pregestational diabetes mellitus resulted in stillbirth. The mother was a 29-year-old Caucasian primigravida female with past medical history of poorly controlled type 2 diabetes managed with metformin prior to pregnancy, prompting admission for glucose management and initiation of insulin at 13 weeks. Baseline hemoglobin A1c was high at 8.0%. Fetal ultrasound at 22 weeks was notable for severe sacral agenesis, bilateral renal pelvis dilatation, single umbilical artery, and pulmonary hypoplasia. Fetal magnetic resonance imaging at 29 weeks showed absent lower two-thirds of the spine with corresponding spinal cord abnormality compatible with type 1 caudal regression syndrome. The mother delivered a male stillborn at 39 and 3/7 weeks. Minimally invasive postmortem magnetic resonance imaging and computed tomography autopsy were performed to confirm clinical findings when family declined conventional autopsy. Etiology of sacral agenesis was attributed to poorly controlled maternal diabetes early in gestation.

**Conclusion:**

Maternal preexisting diabetes is a known risk factor for development of congenital malformations. This rare case of type 1 caudal regression syndrome in a mother with preexisting diabetes with elevated hemoglobin A1c highlights the importance of preconception glycemic control in diabetic women and the utility of fetal magnetic resonance imaging for confirmation of ultrasound findings to permit accurate prognostication. Additionally, minimally invasive postmortem magnetic resonance imaging and computed tomography autopsy can facilitate diagnostic confirmation of clinical findings in perinatal death due to complex congenital anomalies while limiting the emotional burden on bereaved family members who decline conventional autopsy.

## Background

Caudal regression syndrome was first described by Geoffroy Saint-Hilaire and Hohl in 1852 as a rare congenital disease with unclear etiology. The exact cause of caudal regression syndrome remains unknown, although it is likely multifactorial with genetic and environmental factors playing a role. The incidence of caudal regression syndrome has been estimated to affect 1 in 5 per 100,000 live births, but is much more common in infants born to diabetic mothers with 16–22% of cases associated with maternal diabetes [1]. Caudal regression syndrome involves developmental abnormalities of structures derived from the caudal eminence prior to 4 weeks gestation resulting in a wide spectrum of anorectal, spinal, and visceral anomalies [2] and is graded by severity into two main groups or types [3]. Type 1 is more severely affected with the conus ending cephalic to the lower border of the L1 vertebrae, leaving a high-lying club-shaped cord terminus and a sacral deficit at or above the S1 vertebrae. In type 2, the conus ends caudal to the lower border of the L1 vertebrae, and the sacrum tends to be well preserved with identifiable portions of lower vertebrae, although tethering of the spinal cord is more common in those cases. The abrupt spinal cord termination above L1 in type 1 caudal regression syndrome is associated with sacral dysgenesis and usually presents with motor weakness and severe bowel and bladder dysfunction in affected children [3]. Surviving children with type 1 caudal regression syndrome generally exhibit normal intelligence but may have significant motor deficits due to morbidities including dislocation of hips, pelvic deformities, flexion contractures of the knee joints, and talipes equinovarus (clubfeet). Due to multiorgan system involvement in severely affected patients, a multidisciplinary approach to management of type 1 caudal regression syndrome is necessary with delivery at a tertiary care center with access to pediatric subspecialists. For surviving infants, outpatient follow up may include a variety of pediatric specialists, including but not limited to physical medicine and rehabilitation, neurology, neurosurgery, urology, and orthopedics. Associated organ system dysfunction varies but may include respiratory failure and cardiac anomalies in some cases.

This is a case report of a woman with poorly controlled diabetes presenting during early pregnancy ultimately delivering a stillborn infant with type 1 caudal regression syndrome. In this case, early recognition of caudal regression syndrome on routine antenatal ultrasound facilitated confirmation with fetal magnetic resonance imaging (MRI) to characterize the extent of disease and to prognosticate fetal outcome. Following intrauterine fetal demise, minimally invasive computed tomography (CT) and magnetic resonance imaging autopsy confirmed clinical findings while minimizing emotional distress for the bereaved parents who declined conventional autopsy.

## Case presentation

**Fig. 1:**
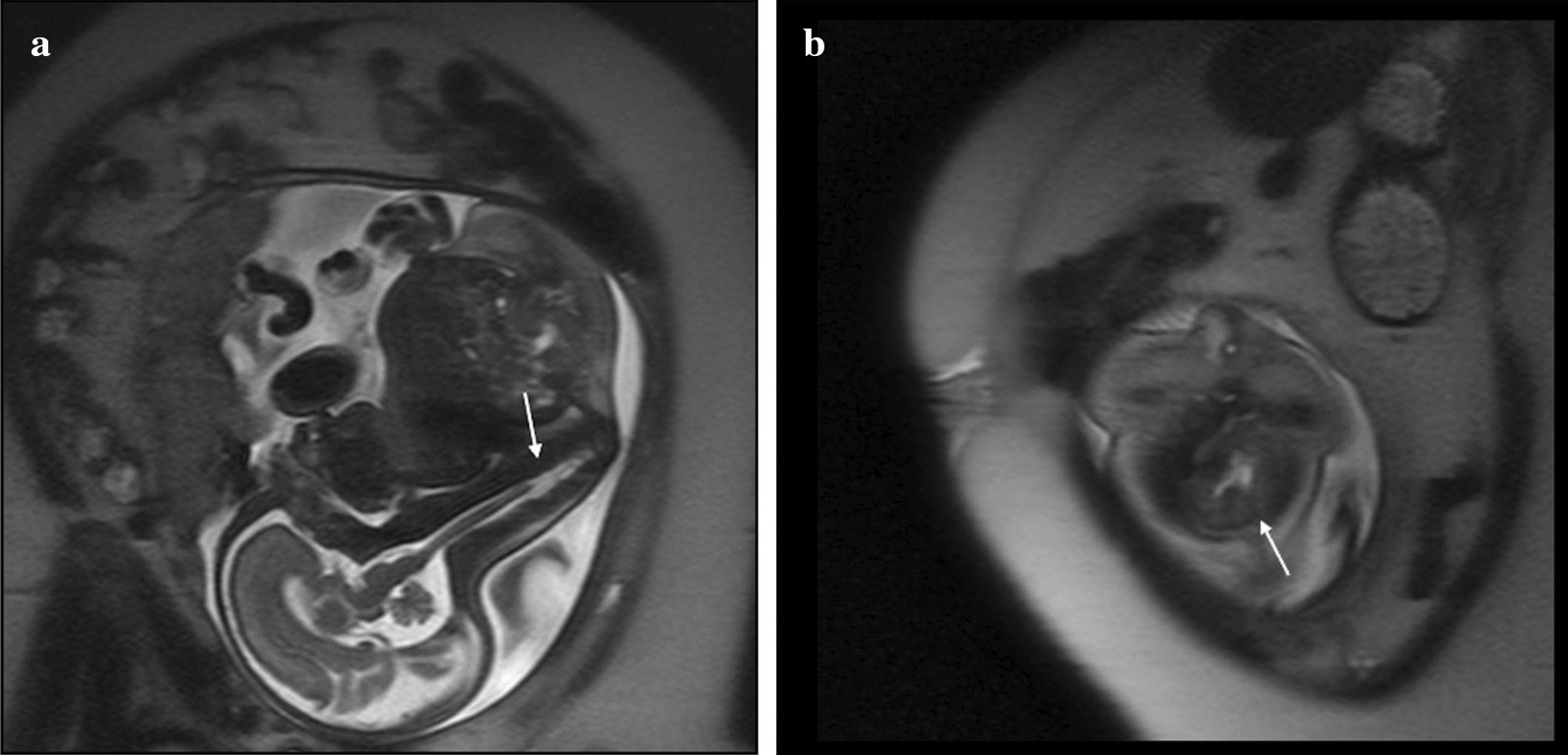
**A** Fetal magnetic resonance imaging at 29 weeks gestation. Sagittal spine T2 Haste sequence on the fetal MRI shows absence of the lower two-thirds of the spine (arrow) and spinal cord abnormality in the fetus. **B** Fetal magnetic resonance imaging at 29 weeks gestation. Coronal spine T2 Haste sequence shows horseshoe kidneys (arrow)

A 29-year-old primigravida Caucasian female with a past medical history significant for short stature, tobacco use, and poorly controlled pregestational type 2 diabetes mellitus (DM) presented to clinic for routine antenatal care. She was first diagnosed with diabetes mellitus at age 22 and managed with metformin prior to pregnancy with a history of poor glycemic control. She was transitioned to glyburide 5 mg twice daily by her obstetrician and subsequently increased to 10 mg twice daily secondary to continued elevated blood glucose levels (fasting ranged from 78 to 113 mg/dl and 2-hour post prandial ranged from 97 to 332 mg/dl). At 13 and 3/7 weeks of pregnancy, the patient was admitted for tighter blood glucose management, initiation of insulin, and a dietician consult to achieve improved control of glucose levels. A baseline hemoglobin A1c was obtained on admission and noted to be high at 8%. Previous maternal hemoglobin A1c levels were not reported. Antenatal fetal ultrasound at 22 weeks was notable for severe sacral agenesis, bilateral renal pelvis dilatation, single umbilical artery, and findings suggestive of pulmonary hypoplasia. The patient was referred to a tertiary care fetal and neonatal medicine center in the USA for further evaluation, and fetal magnetic resonance imaging (MRI) was recommended for confirmatory testing. Fetal MRI at 29 weeks gestation showed absence of the lower two-thirds of the spine with corresponding spinal cord abnormality compatible with type 1 caudal regression syndrome (Fig. 1a) with noted horseshoe kidneys (Fig. 1b). No fetal cardiac anomaly was detected. Fetal anomalies were likely secondary to poorly controlled diabetes during early pregnancy. There was no significant family history.

The mother was counseled extensively by maternal fetal medicine and neonatology that the infant’s prognosis was extremely guarded given the severe spinal cord anomaly and possible pulmonary hypoplasia with substantial risk of intrauterine fetal demise (IUFD), perinatal death, or paraplegia/quadriplegia in a surviving infant. The mother was offered additional confirmatory genetic testing, which she declined. After extensive counseling and discussion of options, which included termination of pregnancy, early induction of labor for provision of comfort care, or continuation of pregnancy to term, the mother opted to continue the pregnancy. She chose to pursue a plan for induction of labor at term (~ 39 weeks) with goal of respiratory resuscitation of the infant and admission to the neonatal intensive care unit (NICU) for stabilization if the infant was viable. She was followed closely throughout the remainder of her pregnancy by maternal–fetal medicine (MFM) and the fetal neonatal medicine center. She continued to return for regular obstetric care for the next 10 weeks with close attention to fetal growth and glucose control. On return obstetric follow up at 37 weeks gestation, the patient’s glucose log showed improved glycemic control with fasting glucose levels < 90 mg/dL and 2-hour postprandial values typically < 120 mg/dL. She was seen by her obstetrician at 39 and 0/7 weeks, at which time there was normal fetal movement and offered induction of labor. She chose to schedule an induction of labor at 39 and 3/7 weeks. Unfortunately, when she presented to labor and delivery for her scheduled induction, antenatal ultrasound showed low amniotic fluid index (AFI) and absent fetal heart tones consistent with intrauterine fetal demise. Labor was medically induced with subsequent vaginal delivery of a stillborn male. Evaluation of the newborn demonstrated normal facies and upper extremities, with normal chest to 2 cm below nipple line and bony spine palpable to approximately the lower two-thirds. No further bony spine was palpable. Feet and legs were present but very small, as was the abdomen, and a left club foot deformity was noted. The baby was small for gestational age with a birth weight of 2070 g, which is less than the first percentile with a *Z* score of − 3.3 according to the Fenton growth chart. Postnatal cytogenetics confirmed a normal male 46 XY karyotype.

**Fig. 2:**
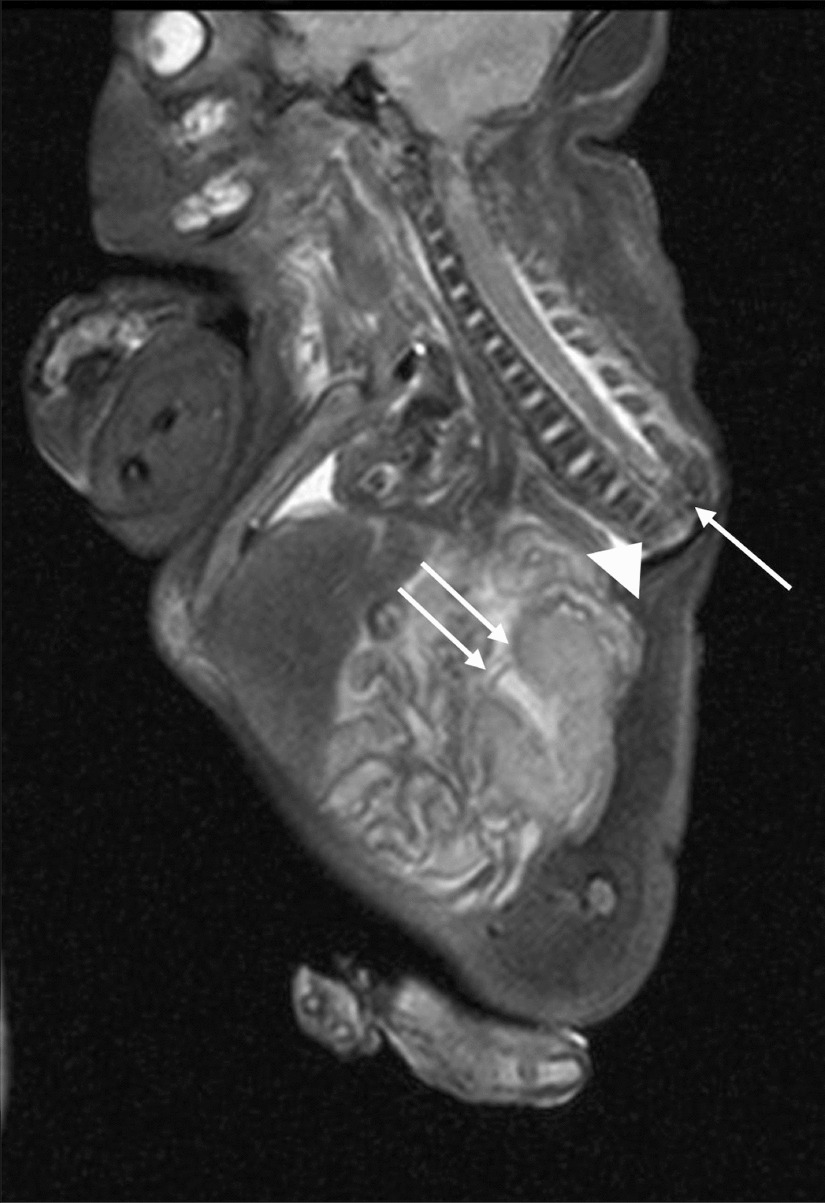
Postmortem MRI autopsy of the stillborn male. T2 WI sequence shows truncated spinal cord (arrow), vertebral column terminating at T7 vertebra (arrow head), and horseshoe kidneys (double arrows)

**Fig. 3:**
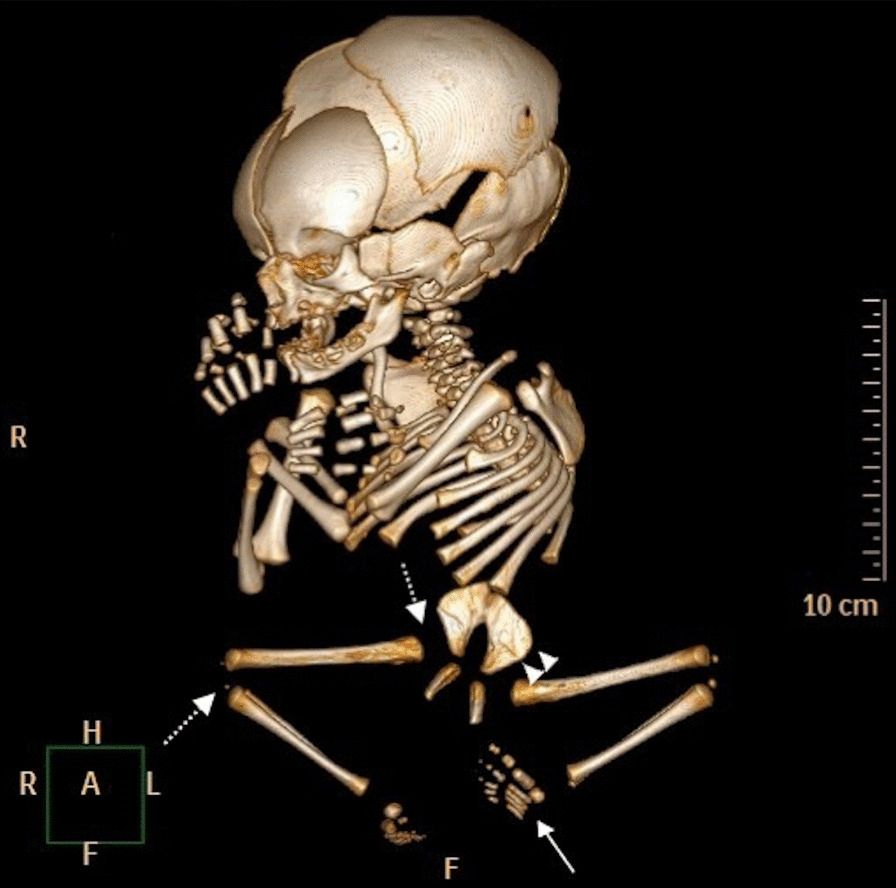
Postmortem computed tomography of the stillborn male. 3D image shows flexion and abduction at the hip with flexion at the knees (dotted arrows), left club foot (arrow), and hypoplastic iliac wings (arrow heads)

The family declined autopsy, so a minimally invasive autopsy was performed. Postmortem MRI confirmed findings of severe caudal agenesis and abrupt cord termination (Fig. 2). Several skeletal abnormalities were visualized on the corresponding CT autopsy (Fig. 3). The infant was in a frog-like position with flexion and abduction at the hips and flexion at the knees. Bilateral iliac wings were hypoplastic and closely approximated at midline secondary to sacral agenesis. Other deformities included a single umbilical artery, horseshoe kidneys (Fig. 2), a left talipes equinovarus deformity, and eight bilateral thoracic ribs (Fig. 3).

## Discussion and conclusion

We present a case of type 1 caudal regression syndrome with a single umbilical artery diagnosed on antenatal ultrasound and fetal MRI, supported by postmortem MRI and CT autopsy findings. Caudal regression syndrome is a rare congenital anomaly that results from abnormal development of the caudal aspect of the spinal cord and vertebral column. The syndrome occurs due to defective neuralization of the mid-posterior axial mesoderm at approximately 4 weeks gestational age [4].

Although Geoffroy Saint-Hilaire and Hohl are credited with first describing the rare congenital disease we now know as caudal regression syndrome, it was Bernard Duhamel who coined the term “caudal regression syndrome” used today. He established the term in the 1960s to encompass a wide spectrum of congenital malformations including sacrococcygeal anomalies, anorectal anomalies, genitourinary anomalies, visceral anomalies, and anomalies of the hind limbs [5]. The most severe form of the spectrum was thought to be sirenomelia, where the lower limbs are fused, resembling a mermaid. This is argued against by Twickler *et al.*, who proposed that sirenomelia is due to an abnormal vascular pattern caused by an single aberrant umbilical artery derived from the vitelline artery, leading to hypoperfusion of the caudal portion of the fetus and severe ischemia [6]. Our presented case had a single umbilical artery on the ultrasound, but the course of the artery was not recognized as aberrant.

Caudal regression syndrome has been associated with poorly controlled maternal diabetes due to the teratogenic effects of the maternal diabetic mileau on the developing embryo, although the precise mechanism of action is not known[7]. Insulin-dependent diabetic women are 200 to 400 times more likely to develop a fetus with caudal regression syndrome than a nondiabetic mother, although the incidence of developing the syndrome in diabetic mothers is decreased with adequate control of diabetes before and during conception, especially during the first few weeks of gestation [1, 7]. In our case, the mother had uncontrolled type 2 diabetes preconception, initially diagnosed 7 years prior to pregnancy.

Based on the position of the conus, patients with caudal regression syndrome are classified into two types [3]. Type 1 is the more severe form of caudal dysgenesis with a club-shaped, high-lying conus that terminates above the lower border of the L1 vertebrae. Type 2 caudal regression syndrome is less severe with a low-lying, tapered conus that terminates below the lower border of the L1 vertebrae [3]. The presented case showed absence of the lower two-thirds of the spine with the caudal cord terminating at the thoracic spine level on the fetal MRI consistent with type 1. It is important to determine corresponding soft tissue and visceral abnormalities associated with caudal regression syndrome to assess severity of disease and guide treatment planning. In our case, the extent of the syndrome was severe, with the fetal and postmortem MRI showing multiple associated abnormalities including decreased number of vertebrae, horseshoe kidneys, and fusion of the iliac wings. No cardiac anomalies or facial dysmorphisms were noted in this small-for-gestational-age infant, although the lower limbs were noted to be small with a talipes equinovarus deformity of the left lower extremity.

The application of postmortem CT and MRI has been increasing in the field of forensic medicine. Meticulous postmortem whole-body analysis in multiple planes with improved image quality is currently feasible with continuous advancements in CT and faster MRI sequences [8]. Routine autopsy was not performed to confirm findings, as this was declined by the parents due to the emotional burden it presented to the family. Postmortem CT and MRI autopsy were therefore used as an alternative as they provided sufficient diagnostic information in a minimally invasive manner. The club-shaped appearance of the spinal cord and the horseshoe kidneys were better visualized on the postmortem MRI (Fig. 2). Postmortem CT demonstrated an accurate vertebral and rib count, showing the presence of seven thoracic rib-bearing vertebrae, and the eighth pair of ribs fusing at midline below the seventh vertebral body (Fig. 3). Postmortem CT with 3D reconstruction showed the baby in a frog-like position (flexion and abduction at the hip, flexion at the knees), with a left club foot and hypoplastic iliac wings fused in the midline (Fig. 3). The mother consented to the case report but declined to comment further on her clinical experience.

Maternal preexisting diabetes is a known risk factor for development of congenital malformations with teratogenic effects on multiple organ systems, although the precise mechanism remains incompletely understood. This rare case of type 1 caudal regression syndrome in a mother with preexisting diabetes with elevated baseline hemoglobin A1c highlights the importance of preconception glycemic control in diabetic women. This case also illustrates the utility of fetal MRI for confirmation of ultrasound findings to permit accurate prognostication, and the effective use of minimally invasive postmortem MRI and CT autopsy as a compelling alternative to nonforensic autopsy to facilitate diagnostic confirmation of clinical findings in perinatal death due to complex congenital anomalies, while limiting the emotional burden on bereaved family members who decline conventional autopsy.

## Data Availability

Not applicable.

## Abbreviations

MFM: Maternal–fetal medicine
OB: Obstetric
MRI: Magnetic resonance imaging
CT: Computed tomography
DM: Diabetes mellitus
IUFD: Intrauterine fetal demise
NICU: Neonatal intensive care unit
AFI: Amniotic fluid index
